# Examining Race-Based and Gender-Based Discrimination, Trust in Providers, and Mental Well-Being Among Black Women

**DOI:** 10.1007/s40615-024-01913-5

**Published:** 2024-02-12

**Authors:** Yendelela L. Cuffee, Portia A. Jackson Preston, Suzanne Akuley, Rachel Jaffe, Sharina Person, Jeroan J. Allison

**Affiliations:** 1 Program in Epidemiology, College of Health Sciences, University of Delaware, 100 Discovery Blvd, Newark, DE 19713 USA; 2 Department of Public Health, California State University, Fullerton, 800 N. State College Drive, KHS-121, Fullerton, CA 92834 USA; 3 University of Delaware, 100 Discovery Blvd, Newark, DE 19713 USA; 4 Population and Quantitative Health Sciences, University of Massachusetts Medical School, 368 Plantation Street, Albert Sherman Center, Worcester, MA 01605 USA

**Keywords:** Black, Women, Discrimination, Provider trust, Mental well-being

## Abstract

**Objectives:**

To examine experiences of discrimination among Black women, and to determine if experiencing race- and gender-based discrimination is associated with mental well-being and trust.

**Methods:**

Data from the TRUST study were used to examine experiences of discrimination among 559 Black women with hypertension receiving healthcare at a safety-net hospital in Birmingham, Alabama. A three-level variable was constructed to combine the race-based and gender-based measures of the Experiences of Discrimination scale. Linear regression was used to examine the association between experiences of discrimination with mental well-being and trust.

**Results:**

Women who reported no experiences of race- or gender-based discrimination were older and reported higher mental well-being scores and greater trust. Fifty-three percent of study participants reported experiencing discrimination. Compared to participants who did not experience race- or gender-based discrimination, participants reporting experiences of race- or gender-based discrimination and those reporting experiencing both race- and gender-based discrimination were more likely to report poorer mental health.

**Conclusion:**

Reported experiences of gender- and/or race-based discrimination in this study were associated with lower mental health scores and less trust in health care providers. Our findings highlight the importance of examining experiences of discrimination among Black women, and the role of discrimination as a stressor and in reducing trust for providers. Incorporating an understanding and acknowledgement of experiences of discrimination into interventions, programs, and during clinical encounters may foster more trusting relationships between providers and patients.

## Introduction

Discrimination has deleterious effects on the physical health and mental well-being of Black women [1]. Experiences of discrimination are defined as unjust treatment or discriminatory intent often predicated by identifying characteristics such as race, ethnicity, social class, gender, and sexuality, and socioeconomic factors. Because experiences of discrimination are self-reported, the determination of whether or not the treatment one receives is discriminatory in nature is reliant on the individual’s perception. These experiences, whether acute or chronic in nature, are a form of social stress and elicit physiological and psychological responses such as the dysregulation of multiple body systems, which compromises the body’s ability to fight disease and increases the risk of morbidity and mortality [2–4].

Previous studies suggest that racial/ethnic minority women who perceived discrimination were more likely to experience poorer health outcomes [5, 6].

Among Black women, experiences of discrimination, attributed to race and gender, may be concomitant stressors, thereby having a greater effect on health and wellness than one factor alone [7]. The double jeopardy hypothesis posits that there is a disadvantage incurred by individuals experiencing discrimination on the basis of membership in multiple marginalized subgroups, such as race and gender, compared to individuals in one disadvantaged group (e.g., Black men) or groups that are not traditionally disadvantaged (e.g., White men) [8]. Crenshaw and Lewis’ theory of intersectionality explains that social identities (e.g., race, gender, ethnicity, and sexuality) intersect and the combined identities may be reflective of a system of either privilege or oppression [9, 10]. In Lewis’ Intersectionality Framework, she explains that individuals possessing multiple highly salient social identities such as race and gender may be more likely to experience socio-structural mistreatment such as discrimination, racism, and sexism, and thus are more likely to have adverse health outcomes [11]. Several studies have revealed that Black women experience some of the highest rates of gender and racial discrimination, and there is a growing body of literature striving to elucidate the relationship between discrimination and physical and mental health outcomes among Black women [10, 12, 13]. A study by Stevens-Watkins et al. found that Black women experiencing sexism were more likely to experience racism, and Black women experiencing sexism are increasingly vulnerable to many lifestyle stressors that could harm psychological well-being [7].

Previous studies indicate that reporting experiences of discrimination may be associated with mistrust towards physician and healthcare providers, particularly among minorities and individuals residing in underserved communities [14]. As explained by Gamble, mistrust in physicians and medical institutions among Blacks is reflective of a long history of experiences of racism inflicted through the exploitation, mistreatment, abuse, and neglect of Blacks at the hands of the medical institutions [15]. Shephard et al. conducted focus groups to explore trust and healthcare among Black women; the study findings revealed that women reporting lower trust were more likely to report experiences of discrimination [16]. Additionally, women who expressed low trust in physicians were less likely to adhere to treatment regimens. A previous analysis of data from the TRUST study indicated that trust in physicians partially mediated the relationship between experiences of discrimination and medication adherence among inner-city Black men and women with hypertension [17]. Given the implications of treatment nonadherence for the effective management of chronic illnesses, further exploration of the relationship between experiences of discrimination and trust is warranted.

There is a critical need to examine how the positionality of Black women at the intersection of marginalized race and gender subgroups, and their identities may uniquely shape their experiences of discrimination and the impact of discrimination on their health and health behaviors. Informed by prior scholarly work on intersectionality of marginalized race and gender on health outcomes, we aim to explore the combination of race- and gender-based discrimination, and the cumulative effects of discrimination on Black women. The primary objective of this study is to examine self-reported experiences of race- and gender-based discrimination among Black women in Birmingham, Alabama, and the association between discrimination and mental well-being and patient-provider trust.

## Methods

The study data were obtained from the TRUST study. TRUST was a subproject within the Alabama Collaboration for Cardiovascular Equality (ACCE) Program which was funded by the National Heart Lung and Blood Institute. The TRUST study was conducted from 2006 to 2008 and included participants recruited from a health system in Birmingham, Alabama. The objectives of the TRUST study were to explore the role of psychosocial, behavioral, and health outcomes among Blacks and Whites with hypertension. The present study was approved by the IRB at the University of Massachusetts Medical School and Penn State University College of Medicine. The details of the TRUST study have been described in detail in previous manuscripts [17, 18].

### Inclusion and Exclusion Criteria

Participants in the TRUST study were age 19 years or older and obtaining care at the health system in Birmingham at the time of data collection. Participants that reported being pregnant were excluded from the study. Informed consent was obtained from all individual participants included in the study. For the present study, participants were eligible if they self-reported their race as African American/Black, reported their gender as female, and responded to the questions about discrimination based on race and gender. A total of 559 Black women were deemed eligible for inclusion in the study.

### Perceived Experiences of Discrimination

Perceived experiences of discrimination were measured using the Experiences of Discrimination (EOD) scale [19]. The EOD scale has six subscales that focus respectively on discrimination based on race or ethnicity or color, weight, socioeconomic status (SES), sexual preference, gender, and religion. Only the questions pertaining to race-based discrimination and gender-based discrimination were included in this study. The race or ethnicity subscale begins with the question, “Have you ever experienced discrimination, were prevented from doing something, hassled or made to feel inferior in any of the following situations because of your race or color?” For the present study, we repeated this question for seven settings: (1) at school, (2) getting a job, (3) getting housing (4), at work (5), at home, (6) seeking medical care, and (7) in public. Each setting received a score of 0–3 based on a response of never, rarely, sometimes, or often (scored respectively). Therefore, the EOD scale ranged from 0 to 21 with a higher score indicating more reported discrimination. The gender subscale begins with the question, “Have you ever experienced discrimination, were prevented from doing something, hassled or made to feel inferior in any of the following situations because of your gender?” For the present study, we repeated this question for the same settings indicated above.

To examine the effects of race-based and gender-based discrimination, we combined the race-based discrimination and gender-based discrimination variables. First, we created a dichotomous yes/no variable for both the gender- and race-based discrimination variables separately. Any participants with a score of 0 indicating no experiences of discrimination were characterized as no; any participants reporting a score of 1–21 indicating experiencing discrimination were categorized as yes. Next, the combined variable was coded as follows: 0 = no reported experiences of race-based discrimination or gender-based discrimination (ND), 1 = experienced either race-based or gender-based discrimination only (ERG), and 2 = experiences race-based and gender-based discrimination (RGD).

### Physical and Mental Functional Status (SF-12)

We assessed physical and mental well-being using the 12-item Short Form Health Survey (SF-12). The SF-12 scale is comprised of two components: physical component score (PCS) and mental component score (MCS). The SF-12 scale assesses eight domains of health: (1) physical functioning; (2) role-physical; (3) bodily pain; (4) general health; (5) vitality; (6) social functioning; (7) role-emotional; and (8) mental health. The scale is normed to the general population, with an average health status score being 50. The scale ranges from 0 to 100, with a higher score indicating better self-reported quality of life.

### Psychosocial Factors (Trust in Physicians)

Trust in physicians was measured using the Hall Trust in Physicians Scale [20]. The Hall Scale has demonstrated excellent internal consistency and reliability (Cronbach alpha = 0.89). The Hall Scale consists of 11 questions that address trust in physicians across five domains of care: (1) honesty, (2) physician competence, (3) caring about the patient’s best interest, (4) confidentiality, and (5) global trust. The Hall Trust in Physicians full scale ranges from 11 to 55, with a higher score indicating greater trust in physicians.

### Covariates

The demographic characteristics examined in the study included education level, annual household income, and age. Annual household income was divided into four categories: < $5000, $5000–$11,999, $12,000–$15,999, >  = $16,000. Education was divided into four categories: (1) less than high school, (2) high school, (3) some college, and (4) college degree.

### Statistical Analyses

We conducted bivariate analysis using ANOVA for continuous variables and the chi-square test for categorical variables, to examine participant characteristics across the main independent variable (level of increasing discrimination). Statistical significance was set at *p* < 0.05. We used linear regression to examine the relationship between categories of race-based and/or gender-based discrimination with participant characteristics. STATA version 17 was used to conduct all statistical analysis (StataCorp, College Station, TX).

## Results

The study population consisted of 559 Black women with hypertension participating in the TRUST study. Characteristics of the study population are presented in Table 1 by the experiences of discrimination categories. The mean age of the study participants was 54 years. Approximately 10% of our sample reported earning a college degree, and 14% of our sample reported a mean annual income of $16,000 or greater. Within our sample, 47% reported that they did not experience discrimination, 39% reported experiencing race- or gender-based discrimination, and 14% reported experiencing both race-based discrimination and gender-based discrimination.

**Table 1:** Characteristics of the study population by category of experiences of discrimination

	Total population (*n* = 559)	No race- or gender-based discrimination (*n* = 260, 47%)	Race- or gender-based discrimination (*n* = 218; 39%)	Gender-based and race-based discrimination (*n* = 81; 14%)	*p*-value
Age, mean (SD)	53.82 (10.12)	54.81 (10.56)	53.58 (10.01)	51.33 (8.49)	0.023
Education, *n* (%)					< 0.001
Less than HS	86 (15.41)	55 (63.95)	26 (30.23)	5 (5.81)	
HS	78 (13.98)	35 (44.87)	33 (42.31)	10 (12.82)	
Some college	341 (61.11)	162 (47.51)	130 (38.12)	49 (14.37)	
College degree	53 (9.50)	8 (15.09)	29 (54.72)	16 (30.19)	
Annual household income, *n* (%)					0.183
< $5000	151 (29.15)	75 (49.67)	58 (38.41)	18 (11.92)	
$5000–11,999	194 (37.45)	97 (50.00)	67 (34.54)	30 (15.46)	
$12,000–15,999	98 (18.92)	41 (41.84)	42 (42.86)	15 (15.31)	
> = 16,000	75 (14.48)	25 (33.33)	35 (46.67)	15 (20.00)	
PCS, mean (SD)^a^	36.69 (11.28)	37.90 (11.19)	35.96 (11.21)	34.74 (11.46)	0.044
MCS, mean (SD)^a^	44.84 (12.55)	46.33 (12.68)	44.14 (12.30)	41.92 (12.28)	0.013
Trust, mean (SD)^b^	38.61 (8.21)	41.12 (7.01)	37.49 (8.32)	33.53 (8.58)	< 0.001

^a^Physical (PCS) and Mental (MCS) Component Scores were measured using the SF-12 Survey and ^b^Trust in Physicians was measured using the Hall Trust in Physician Scale

We found statistically significant relationships between experiences of discrimination and mental composite score, physical composite score, trust, age, and education. Mental composite scores were lowest among those who reported experiences of both gender-based discrimination and race-based discrimination (ND 46.33, ERG 44.14, RGD 41.92, *p* = 0.013). Similarly, trust scores were lowest among those reporting experiences of both gender-based discrimination and race-based discrimination (ND 41.12, ERG 37.49, RGD 33.53, *p* < 0.001). Participants reporting no experience of race- or gender-based discrimination were older compared to other participants (ND 54.81, ERG 53.58, RGD 51.33, *p* = 0.023), reported better physical health (ND 37.90, ERG 35.96, RCG 34.74, *p* = 0.044), and were more likely to have less than a high school education (ND 63.95%, ERG 30.23%, RGD 5.81%, *p* < 0.001). We did not detect a statistically significant association with experiences of discrimination and income.

### Trust in Physicians

Table 2 provides a summary of the association between discrimination (no discrimination, either race-based or gender-based discrimination, gender-based and race-based discrimination) and trust in physician adjusting for age, education, income, and physical and mental well-being. We found a statistically significant association between reporting having experienced race- or gender-based discrimination (*β* =  − 2.810, CI − 4.282, − 1.339, *p* < 0.001), and race- and gender-based discrimination (*β* =  − 5.860, CI − 7.895, − 3.824, *p* < 0.001) with trust in providers. Additionally, with other factors held constant, the following factors were found to have a statistically significant relationship with trust. The factors were age (*β* = 0.069, CI 0.002, 0.137, *p* = 0.045), physical (*β* = 0.072, CI 0.019, 0.132, *p* = 0.019) and mental component scores (*β* = 0.102, CI 0.046, 0.159, *p* < 0.001), and having a high school education (*β* = 2.602, CI 0.168, 5.036, *p* = 0.036) or college degree (*β* =  − 2.892, CI − 5.685, − 0.099, *p* = 0.042).

**Table 2:** Adjusted association of race- and gender-based discrimination with trust in providers among 557 Black women participating in the TRUST study, 2007–2008

	Coefficient (*β*)	Confidence interval	*p*-value
Discrimination category (ref = no race- or gender-based)			
Race- or gender-based	− 2.810	− 4.282, − 1.339	< 0.001
Race- and gender-based	− 5.860	− 7.895, − 3.824	< 0.001
Age	0.069	0.002, 0.137	0.045
Education (ref = < high school)			
High school	2.602	0.168, 5.036	0.036
Some college	0.435	− 1.482, 2.352	0.656
College degree	− 2.892	− 5.685, − 0.099	0.042
Annual household income (ref = < $5,000)			
$5000–$11,999	− 0.332	− 1.962, 1.299	0.690
$12,000–$15,999	− 1.013	− 2.987, 0.962	0.314
$ > = 16,000	− 0.795	− 2.998, 1.407	0.478
Physical Component Score^a^	0.072	0.019, 0.132	0.019
Mental Component Score^b^	0.102	0.046, 0.159	< 0.001

Reporting never experiencing race- or gender-based discrimination was the reference group. ^a^Physical and Mental Component Scores were measured using the SF-12 Survey and ^b^Trust was measured using the Hall Trust in Physician Scale

### Mental Well-Being

To further explore the documented association between discrimination and mental well-being, we used linear regression to examine discrimination and well-being, adjusting for demographic variables, physical well-being, and trust. Table 3 provides a summary of the association between discrimination (no discrimination, either race-based or gender-based discrimination, gender-based and race-based discrimination) and mental well-being adjusting for age, education, income, and physical well-being. We did not detect statistically significant association between reporting having experienced race-based or gender-based discrimination (*β* =  − 2.140, CI − 4.413, 0.134, *p* = 0.065). Race- and gender-based discrimination were significantly associated with mental well-being (*β* =  − 3.802, CI − 6.939, − 0.665, *p* = 0.018). With other factors being held constant, our findings revealed a statistically significant relationship between age (*β* = 0.295, CI 0.194, 0.397, *p* < 0.001), some college (*β* = 3.079, CI − 0.121, 6.038, *p* = 0.041), and a household income of $12,000–$15,999 (*β* = 3.641, CI 0.598, 6.038, *p* = 0.019) and $16,000 or more (*β* = 6.155, CI 2.785, 9.526, *p* < 0.001) with mental well-being.

**Table 3:** Adjusted association of race- and gender-based discrimination with mental well-being among 557 Black women participating in the TRUST study, 2007–2008

	Coefficient	Confidence interval	*p*-value
Discrimination category (ref = no race- or gender-based)			
Race- or gender-based	− 2.140	− 4.413, 0.134	0.065
Race- and gender-based	− 3.802	− 6.939, − 0.665	0.018
Age	0.295	0.194, 0.397	< 0.001
Education (ref = < high school)			
High school	1.030	− 2.741, 4.801	0.592
Some college	3.079	0.121, 6.038	0.041
College degree	2.591	− 1.731, 6.914	0.239
Annual household income (ref = < $5000)			
$5000–$11,999	0.118	− 2.409, 2.645	0.927
$12,000–$15,999	3.641	0.598, 6.685	0.019
$ > = 16,000	6.155	2.785, 9.526	< 0.001
Physical Component Score^a^	0.085	− 0.008, 0.178	0.074

Reporting never experiencing race- or gender-based discrimination was the reference group. ^a^Physical and Mental Component Scores were measured using the SF-12 Survey and ^b^Trust was measured using the Hall Trust in Physician Scale

## Discussion

The objective of this study was to determine the impact of perceived discrimination on mental well-being and among Black women with hypertension. Approximately 53% of our participants reported experiencing discrimination, and slightly less than half of our participants reported they did not experience race- or gender-based discrimination. Our approach for examining discrimination within this group of women is unique in that participants reported lifetime experiences of discrimination and our discrimination measure assessed the impact of the experiences in an additive manner, specifically individuals experiencing no discrimination, individuals experiencing at least one form of discrimination, and individuals experiencing both forms of discrimination. Previous studies suggest that the effect of race-based and gender-based discrimination may be compounding, and it is difficult to isolate the rationale behind experiences of discrimination as solely driven by race or gender given the intersectional positionality of Black women [21].

The Black women participating in the TRUST study that reported experiences of any form of discrimination reported lower mental well-being scores compared to women who reported never experiencing discrimination. Our findings were consistent with a study conducted by Schulz et al. (2006), which found a positive association between instances of experiencing discrimination and depression among Black women [22]. A meta-analysis conducted by Pascoe et al. (2009) indicated perceived discrimination was often associated with multiple dimensions of mental well-being, as well as states of negative affect such as depression, psychological distress, and clinical levels of mental illness [23]. Stevens-Watkins et al. (2019) surveyed African American women and found that “multiple levels of oppression,” including being Black, female, and of lower socioeconomic status, increased one’s susceptibility to psychological distress; they also found that increased risk for psychological distress was correlated with personal injury and illness among Black women [7].

In the present study, experiences of discrimination were associated with lower trust in health care providers. Relative to women who reported no race- or gender-based discrimination, those who reported race- or gender-based discrimination and race- and gender-based discrimination had lower trust in their health care providers. Our findings are similar to the results of the LaVeist et al. (2000) study of African American and White cardiac patients seeking care from three hospitals in Maryland [24]. Participants were asked about satisfaction with care and racism in the medical system. The authors found that African Americans that perceived racism were similarly more likely to harbor mistrust towards the medical system and were less satisfied with health care [25]. Furthermore, a study by Sutton et al. indicated Black women’s mistrust of providers has been associated with perceptions of experiencing discrimination from healthcare providers [26].

Lastly, the findings of our study revealed that education may be an important correlate of discrimination. Women that attained a college education, attended some college, or completed high school or a GED were more likely to report experiencing discrimination compared to women that did not complete high school. A study by Hudson et al. examined socioeconomic position and racial discrimination among Black women; among their participants, they did not observe any interactions between socioeconomic position and race-based discrimination [27]. This may be attributed to Black adults with a higher SES being more likely to live in integrated communities and work in diverse settings, which may increase their likelihood of experiencing stressors such as discrimination [27, 28]. Hudson et al. also noted that Black women’s experiences of race-based discrimination may be underestimated. Conversely, a study by Dailey et al. examined socioeconomic position and perceptions of racial discrimination among 1239 Black and White women and found that women with higher levels of socioeconomic disadvantages were less likely to report experiences of discrimination [29].

### Limitations and Strengths

While our study provides useful insights into the experiences of discrimination among Black women in America, our study is not without limitations. First, we assessed the relationship between discrimination and measures of mental and physical well-being using cross-sectional data; thus, we are unable to explore causal relationships or disentangle the complex relationship between discrimination, health outcomes, and trust. Discrimination data for this study was obtained from patient self-report and may be subject to desirability bias. However, measures of discrimination often rely upon an individual’s self-report of perceived discriminatory experiences [30, 31]. It should also be noted that reported experiences of discrimination were low among our study participants; the average score for experiencing gender-based discrimination is 2.80 and race-based discrimination 1.81; however, our rates are similar to other studies exploring discrimination using the Experiences of Discrimination scale [32]. Our study did not capture experiences of discrimination due to factors other than race or gender, nor did it compare experiences of Black women to other groups. Our sample focuses on the experiences of Black women in Alabama in 2008 and is not representative of all Black women. Therefore, the findings may not be generalizable to other regions, racial and ethnic groups, and individuals of other gender identities. It is, however, important to note the setting in which the TRUST study was conducted, Birmingham, Alabama, a historic city, which played a pivotal role during the Civil Rights Movement. Based upon the average age of our participants at the time of the study, some of the participants may have lived during Civil Rights Movement and may have a unique historical and personal awareness of mistreatment and discriminatory experiences inclusive of that time period. Our analysis cannot account for the social and political changes that have taken place since the study data was collected that may impact the reporting of discrimination and trust. While the TRUST study data was collected in the early 2000, it has been used to examine important social constructs such as happiness, John Henryism, and discrimination that continue to impact the health and well-being of Blacks living in America today. However, from the TRUST data, we can glean important insights into the experiences of Black Americans from the information and experiences shared by the TRUST study participants.

### Public Health Implications

We explored the role of discrimination as a correlate of trust and mental well-being. Our findings indicated that among Black women, experiences of race-based and gender-based discrimination were associated with lower mental well-being and less trust in providers. Our findings highlight the need for future studies to further disentangle the relationship between discrimination, health outcomes, and health behaviors. We also recognize the importance of continuing to study discrimination as experienced by individuals with multiple marginalized identities, and how personal identity may influence an individual’s response to discrimination. This may provide insights to the coping strategies and support systems that are employed in response to experiences of discrimination, which might be integrated into future programs and studies.

Individuals experiencing discrimination may be less likely to trust healthcare providers or the healthcare system, report satisfaction with healthcare, or adhere to prescribed medical recommendations or medications [33]. Therefore, approaches such as interventions and programs that promote patient-provider communication, encourage cultural competence, and promote the importance of the patients’ needs and patient voice may be effective in bolstering trusting relationships between patients and providers. In the clinical setting, there is a need to center patients’ previous experiences in healthcare by acknowledging past missteps, providing opportunities to explore experiences of discrimination, and addressing concerns about medications and treatment plans. Integrating of these aspects into clinical encounters may bolster trust, reduce resistance to health information, and contribute to better health outcomes.

## Data Availability

Data is available by requesting permission from the TRUST study PI.
